# A Link Between Gut Microbiota and Schizophrenia

**DOI:** 10.1192/j.eurpsy.2022.1972

**Published:** 2022-09-01

**Authors:** J. Sá Couto, M. Pão Trigo, B. Da Luz, J. Rodrigues, T. Ventura Gil

**Affiliations:** Centro Hospitalar Universitário do Algarve, Departamento De Psiquiatria E Saúde Mental, Unidade De Faro, Faro, Portugal

**Keywords:** Gut microbiota, schizophrénia, Pathogenesis

## Abstract

**Introduction:**

Microorganisms distributed in our tissues and fluids make up the human microbiota. During our lifetime, gastrointestinal microbiota acts as an important modulator of brain development and, in turn, adult behavior and health. Immune response may be triggered by gut microbiota, releasing mediators that penetrate the blood-brain barrier (BBB).

**Objectives:**

Understanding if gut microbiota can influence schizophrenia pathogenesis. Clarifying how gut microbiota can influence schizophrenia treatment, and vice-versa.

**Methods:**

*PubMed* database search, with *“gut microbiota and schizophrenia”* keyword expression. Eight articles published in the last ten years were selected among the most recent best match results. Reference lists of articles were reviewed to identify additional articles.

**Results:**

There could be an association between the development of gut microbiota starting during pregnancy and schizophrenia pathogenesis, through an immune-mediated process. Schwarz *et al.* (2018) investigated the differences in faecal microbiota between individuals with first-episode psychosis and controls. They found psychotic patients to have an increased amount of Lactobacillus bacteria. Yuan *et al.* (2018) studied microbiota changes in patients with schizophrenia, before and after treatment. Individuals diagnosed with schizophrenia had less faecal Bifidobacterium, Escherichia coli and Lactobacillus. After treatment with risperidone, there was a significant increase in the amount of fecal Bifidobacterium and E. Coli.

**Conclusions:**

Microorganisms living inside our gastrointestinal tract are vital for proper central nervous system (CNS) development. Patients with schizophrenia have anomalies in the composition of the microbiota. It remains unclear if microbiota changes after treatment further influence the course of the disease.

**Disclosure:**

No significant relationships.

